# The burden of radiation exposure in congenital heart disease: the Italian cohort profile and bioresource collection in HARMONIC project

**DOI:** 10.1186/s13052-024-01663-4

**Published:** 2024-05-17

**Authors:** Jonica Campolo, Giuseppe Annoni, Gabriele Vignati, Alessio Peretti, Marco Papa, Paola Enrica Colombo, Gaia Muti, Marina Parolini, Andrea Borghini, Marzia Giaccardi, Lamia Ait-Alì, Eugenio Picano, Maria Grazia Andreassi

**Affiliations:** 1 CNR Institute of Clinical Physiology, ASST Grande Ospedale Metropolitano Niguarda, Piazza Ospedale Maggiore 3, 20162 Milan, Italy; 2 Pediatric Cardiology, Regina Margherita Children’s Hospital, Turin, Italy; 3 Pediatric Cardiology, ASST Grande Ospedale Metropolitano Niguarda, Milan, Italy; 4 Medical Physics, ASST Grande Ospedale Metropolitano Niguarda, Milan, Italy; 5 CNR Institute of Clinical Physiology, Pisa, Italy; 6 Department of Internal Medicine, Electrophysiology Unit, Santa Maria Annunziata Hospital, Florence, Italy

**Keywords:** Congenital heart disease, Cardiac catheterization, Ionizing radiation

## Abstract

**Background:**

The European-funded Health Effects of Cardiac Fluoroscopy and Modern Radiotherapy in Pediatrics (HARMONIC) project aims to improve knowledge on the effects of medical exposure to ionizing radiation (IR) received during childhood. One of its objectives is to build a consolidated European cohort of pediatric patients who have undergone cardiac catheterization (Cath) procedures, with the goal of enhancing the assessment of long-term radiation-associated cancer risk.

The purpose of our study is to provide a detailed description of the Italian cohort contributing to the HARMONIC project, including an analysis of cumulative IR exposure, reduction trend over the years and an overview of the prospective collection of biological samples for research in this vulnerable population.

**Methods:**

In a single-center retrospective cohort study, a total of 584 patients (323 males) with a median age of 6 (2–13) years, referred at the Pediatric Cardiology in Niguarda Hospital from January 2015 to October 2023, were included. Biological specimens from a subset of 60 patients were prospectively collected for biobanking at baseline, immediately post-procedure and after 12 months.

**Results:**

Two hundred fifty-nine (44%) patients were under 1 year old at their first procedure. The median KAP/weight was 0.09 Gy·cm^2^/kg (IQR: 0.03–0.20), and the median fluoroscopy time was 8.10 min (IQR: 4.00–16.25). KAP/weight ratio showed a positive correlation with the fluoroscopy time (Spearman’s rho = 0.679, *p* < 0.001). Significant dose reduction was observed either after implementation of an upgraded technology system and a radiation training among staff. The Italian cohort includes 1858 different types of specimens for Harmonic biobank, including blood, plasma, serum, clot, cell pellet/lymphocytes, saliva.

**Conclusions:**

In the Italian Harmonic cohort, radiation dose in cardiac catheterization varies by age and procedure type. An institution’s radiological protection strategy has contributed to a reduction in radiation dose over time. Biological samples provide a valuable resource for future research, offering an opportunity to identify potential early biomarkers for health surveillance and personalized risk assessment.

## Background

Congenital heart disease (CHD) is one of the most prevalent birth defects among humans, Impacting roughly 1% of all births [1]

Epidemiological studies reveal a notably heightened susceptibility to various types of cancer, including leukemia, tumors of the central nervous system, tumors of the sympathetic nervous system, and soft tissue sarcomas among individuals diagnosed with CHD compared to the general population [2].

The origins and the risk factors contributing to the acquired cancer risk in CHD remain largely elusive, and exposure to ionizing radiation (IR) from X-ray examinations and fluoroscopically guided cardiac catheterization (cath) procedures is considered a potentially relevant factor [3–9], particularly in patients receiving repetitive and cumulative radiation doses during early life [10].

However, large and well-designed epidemiological studies with an interdisciplinary and global approach are imperative to establish the connection between radiation exposure from cardiac procedures and the onset of cancer [11].

A recent five-year multicenter project, entitled "The Health Effects of Cardiac Fluoroscopy and Modern Radiotherapy in Pediatrics (HARMONIC)" (2019–2024), was funded by the European Commission. One of its main objectives is to build a consolidated European cohort comprising patients who underwent cath procedures across seven countries (Belgium, France, Italy, Germany, Norway, Spain, and the UK). This initiative aims to provide substantial insights into the risk of cancer associated with childhood IR exposure, with statistical power strengthened by the collaboration of multiple nations, a feat unattainable through single national studies [12].

Another potent strategy is the establishment of a biobank to provide a mechanistic understanding of radiation-induced adverse biological effects and identify potential early biomarkers that could facilitate health surveillance and personalized risk assessment [2, 13].

The aim of this study is to provide a comprehensive description of the Italian cohort of CHD patients contributing to the HARMONIC project. This description includes a detailed analysis of cumulative IR exposure, trend over the years, and an overview of the collected prospective biobank designed for research purposes within this vulnerable population.

## Methods

### Study population

**Fig. 1:**
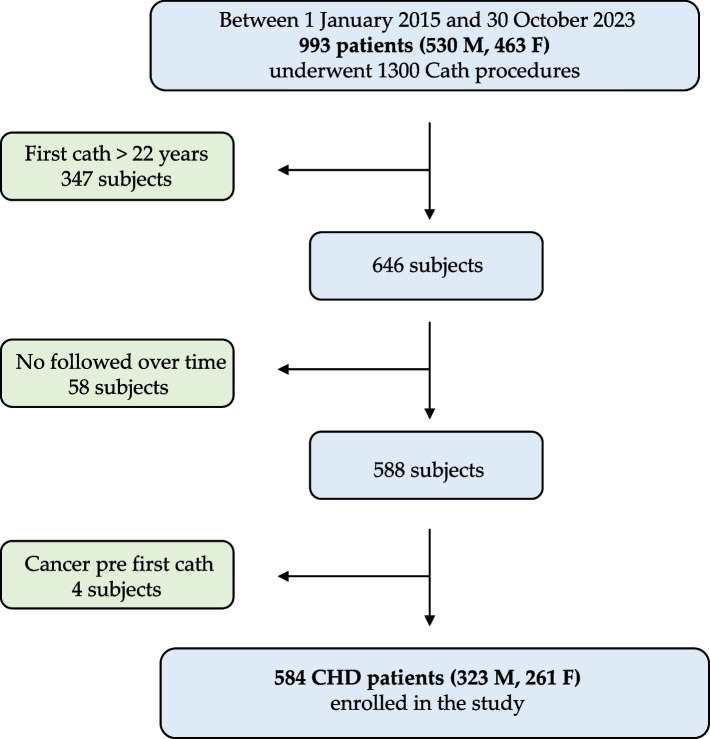
Flowchart of patients’ selection and enrollment

As part of the ongoing HARMONIC project [12], we presented an Italian national cohort comprising patients with CHD who had not been diagnosed with cancer and had undergone at least one cardiac catheterization. In this single-center, observational, retrospective study, all patients referred for a cath procedure at the Pediatric Cardiology in Niguarda Hospital between 1 January 2015 and 30 October 2023 were screened for inclusion. Hospital medical records for 993 patients were obtained from the pediatric cardiology department. Patients were considered eligible for inclusion if they underwent at least one cardiac cath procedure at an age < 22 years. Based on the criteria set by HARMONIC [12], patients were excluded if they lacked follow-up data, or had received a cancer diagnosis before the first cath procedure. The flowchart in Fig. 1 shows the exclusion and inclusion of patients in this study. A total of 584 patients (261 females, 323 males) with 1181 cath procedures were included in the cohort.

The study was approved by the Institutional Ethical Committee (Study Protocol n. 605–112019).

### Data collection

We retrieved clinical, demographics and procedural information for each patient from the electronic hospital's archive.

Dose cath indicators, including Kerma Area Product (KAP, Gy·cm^2^) and Fluoroscopic time (FT, min) were recorded for each patient during the procedure by the system’s radiation dosimeter coupled to the angiography imaging system.

Until August 2010, cath procedures were performed using a Philips Integris H5000 system (Philips Healthcare). Subsequently, the hospital upgraded its equipment with a GE Innova 2100-IQ system (General Electric Healthcare) for diagnostic and interventional procedures and Philips Integris Allura, specifically for electrophysiology procedures.

In September 2012, a radiation dose-reduction program was also implemented in the catheterization laboratory. All medical and technical staff participated in a training course focused on optimizing doses in fluoroscopy-guided procedures, covering aspect such as fluoroscopy levels, frame rates and geometrical parameters.

Additionally, hospital medical records were also used to retrive information on other high dose procedures, including computed tomography (CT), nuclear medicine (NM) scans and whole body or cardiac positron emission tomography-computed tomography (PET-CT).

Radiation exposure was extracted from imaging reports by collecting Dose‐Length Product (DLP, mGy·cm) for CT and PET-CT, and administered activity (MBq, Megabecquerel) of the tracer for NM scans.

### Biologic sample processing

Biological samples were collected in a subgroup of these patients who underwent diagnostic and/or therapeutic cardiac catheterization procedures, at three-time points: immediately before, immediately after the procedure and approximately 1 year afterward. Informed consent was obtained from all subjects or their parents.

Briefly, a maximum of 5 mL of blood sample was collected in a vacutainer tube containing EDTA K2, one clot activator serum separation tube, and one BD Vacutainer® CPT™ tube for isolation of lymphocytes. Within two hours post collection, blood samples were centrifuged according to standard operating procedures to obtain plasma, serum, and lymphocytes. Aliquots of each fraction were prepared and immediately stored at − 80 °C for future biomarker analysis, as previously reported [13]. Saliva samples were also acquired and divided into aliquots.

### Statistical analysis

Categorical variables are expressed as frequencies and percentages, while continuous variables as median and interquartile range (IQR): 25th and 75th percentiles. Nonparametric tests were chosen due to the skewed distribution. The comparison between two groups was assessed using the Mann–Whitney U test and correlations between continuous variables were tested by using Spearman’s rho value.

Patients were stratified into age categories at the procedures: newborn (0–30 days), infants (1–12 months), 1–5 years, 6–10 years, 11–15 years; and > 15 years.

According to a training course, we compared radiation measures obtained using GE Innova 2100-IQ between the pre-course period (before training) and the post-course period (after training). Statistical analyses were performed using SPSS ver. 24.0 software package (IBM SPSS, New York, USA). *P*-value < 0.05 was considered as statistically significant.

## Results

### Patients’ cohort

The baseline and clinical characteristics of patients are detailed in Table 1. Among CHD patients, 1181 diagnostic and/or therapeutic cardiac catheterization procedures were conducted, averaging 2.02 procedures per person.

**Table 1:** Baseline and clinical characteristics of the Italian cohort

Characteristics	Patient Cohort (*N* = 584)
Age at the enrollment, median (IQR)	6 (2–13) years
Genetic/syndromic CHD, N.(%)	67 (12)
*Gender*	
Female (N.)	261 (45)
Male (N.)	323 (55)
Age at the first cath exposure, median (IQR)	1 (0–8) years
N. of diagnostic procedures (%)	664 (56)
N. of interventional procedures (%)	517 (44)
*N. of cath procedures/patient*	
1, N. (%)	354 (61)
2, N. (%)	94 (16)
3, N. (%)	61 (10)
> 3, N. (%)	75 (13)
Total number of other high dose examinations	300

*CHD* Congenital heart diseases, *IQR* Interquartile range

The median age at first exposure was 1 year (IQR: 0–8). Two hundred fifty-nine (44%) patients underwent their first procedure before reaching 1 year of age, and 109 patients (19%) underwent catheterization within hours or days after birth.

Additionally, 300 other high dose procedures (CT, PET-CT, and NM scan) were also performed in 148 CHD patients.

### Radiation exposure data

Dosimetric data were adequately collected for 94% of the total catheterization procedures.

The median KAP/weight was 0.09 Gy·cm^2^/kg (IQR: 0.03–0.20), and the median FT was 8.10 min (IQR: 4.00–16.25). KAP/weight showed a positive correlation with FT (Spearman’s rho = 0.679, *p* < 0.001).

Significant differences were observed in the KAP/weight and FT between diagnostic and therapeutic procedures (median KAP/weight 0.08 Gy·cm^2^/kg IQR: 0.03–0.17 versus 0.10 Gy·cm^2^/kg IQR: 0.05–0.25, *p* < 0.001, and median FT 6.00 min, IQR: 3.00–13.02 versus 11.22 min IQR: 6.32–23.09, *p* < 0.001, respectively).

Dosimetric parameters based on the individual procedure type are presented in Table 2.

**Table 2:** KAP/weight and fluoroscopy time in different types of procedures

Procedure type	KAP/weight Gy.cm^2^/kg	FT min
**Diagnostic (*N* = 664)**		
Cardiac diagnostic catheterism (*N* = 505)	0.08 (0.03–0.18)	7.00 (3.32–15.00)
Coronary angiography (*N* = 55)	0.09 (0.05–0.20)	5.50 (3.04–10.00)
Endomyocardial heart biopsy (*N* = 67)	0.01 (0.01–0.02)	2.36 (1.41–4.26)
Other diagnostic procedures (*N* = 37)	0.06 (0.01–0.46)	4.12 (1.03–8.06)
**Interventional (*N* = 517)**		
Atrial septal defect occlusion (*N* = 55)	0.05 (0.02–0.12)	7.00 (4.19–11.00)
Ventricular septal defect occlusion (*N* = 10)	0.42 (0.09–0.73)	23.82 (7.33–39.33)
PDA occlusion (*N* = 75)	0.05 (0.02–0.13)	7.55 (5.07–11.15)
Atrial septostomy (*N* = 57)	0.08 (0.03–0.16)	7.52 (4.48–17.41)
Balloon pulmonary valvuloplasty (*N* = 72)	0.09 (0.06–0.21)	14.56 (9.60–26.20)
Balloon aortic valvuloplasty (*N* = 9)	0.10 (0.06–0.63)	17.37 (9.65–34.50)
Pulmonary artery balloon/stent (*N* = 101)	0.29 (0.11–0.74)	32.50 (13.00–49.01)
Coarctation of aorta repair balloon/stent (*N* = 74)	0.13 (0.07–0.24)	11.00 (7.45–16.52)
Electrophysiology procedures (*N* = 35)	0.05 (0.01–0.26)	7.50 (1.88–20.00)
Other interventional procedures (*N* = 29)	0.14 (0.09–0.36)	13.00 (1.10–34.70)

*KAP* Kerma area product, *FT* Fluoroscopic time, *PDA* Patent ductus arterious

**Fig. 2:**
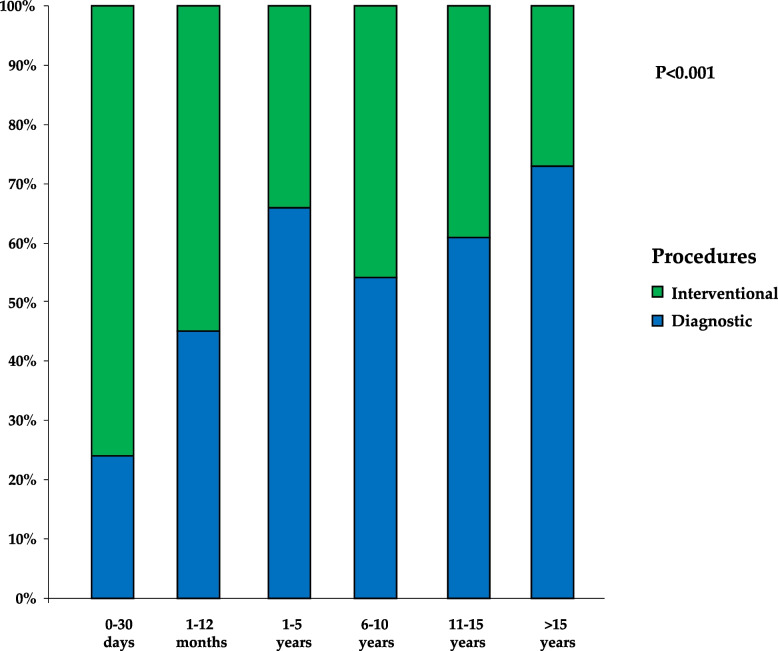
Proportion of diagnostic and therapeutic procedures in the CHD cohort categorized by age groups. P refers to the Chi-square test between procedures type and age category group

**Fig. 3:**
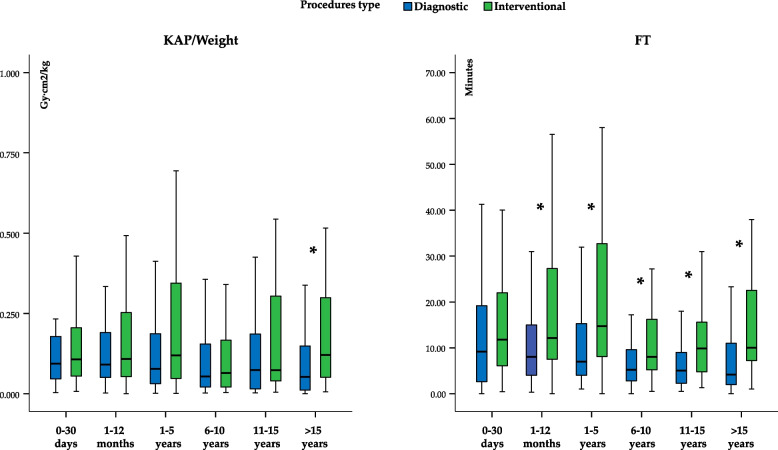
Differences in KAP/weight and FT values between diagnostic and interventional procedure at each age group. P refers to the Mann–Whitney U test for KAP/Weight and FT between procedures type in each age category group. *P < 0.05 adjusted by multiple comparisons

When patients were categorized into age subgroups, a statistically significant difference (*P* < 0.001) was observed in the distribution between diagnostic and therapeutic procedures. The proportion of interventional cases decreased progressively with advancing years (Fig. 2). The radiation dose, expressed as KAP/weight, was significantly higher in therapeutic procedures compared to diagnostic ones in the age group > 15 years (*P* < 0.05 adjusted by multiple comparisons). Conversely, FT displayed significant differences between procedures in all age groups, except in the newborn group (Fig. 3).

### Changes in radiation exposure by different equipment and training course

**Fig. 4:**
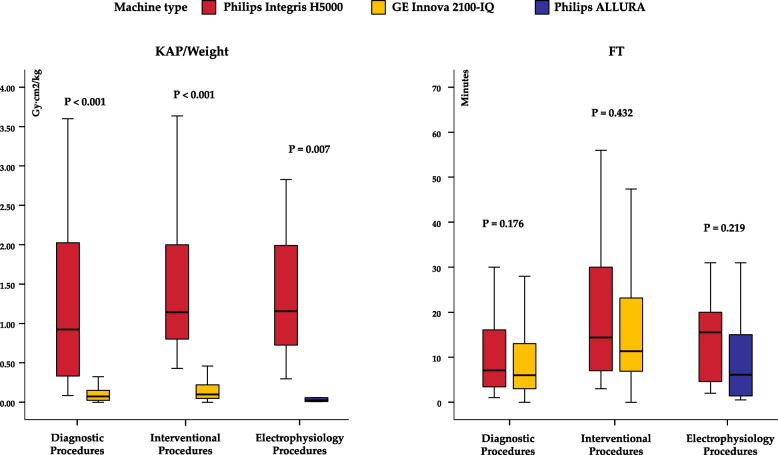
Boxplots of KAP/weight and FT in diagnostic procedures, interventional procedures, and electrophysiology studies according to different equipments. P refers to the Mann–Whitney U test for KAP/Weight and FT between machine type in diagnostic, interventional and electrophysiology procedures group

The comparison of exposure data reveals a significant dose reduction with the upgraded technology system, transitioning from Philips Integris H5000 (N. of procedures = 86) to GE Innova 2100-IQ (N. of procedures = 1008) or Philips Integris ALLURA (*N* = 20). The KAP/weight exhibited a significant decrease in the upgraded equipment compared to the previous system (Fig. 4). In contrast, no significant difference was observed for FT parameters between equipments.

**Fig. 5:**
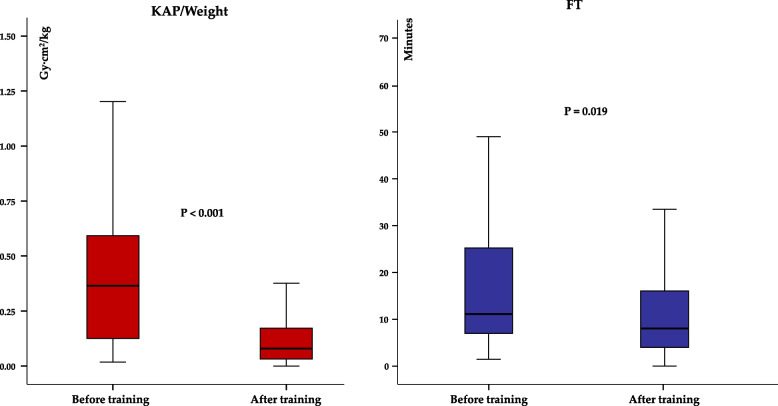
Comparison of KAP/weight and FT before and after the training course. P refers to the Mann–Whitney U test for KAP/Weight and FT between training group

Moreover, we compared dose parameters before and after the training course carried out in September 2012. The analysis highlighted statistically significant differences in KAP/weight and FT between the two periods, as depicted in Fig. 5.

### Other imaging radiation procedures

**Fig. 6:**
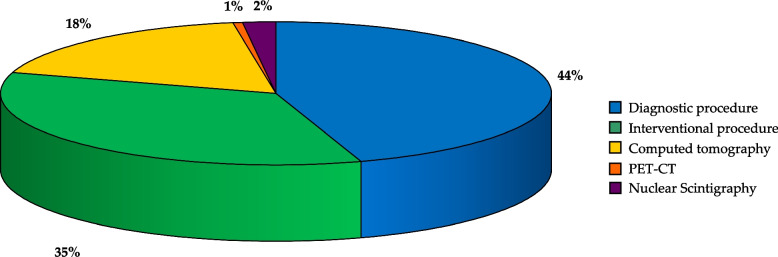
Distribution of ionizing radiation examination in the Italian CHD cohort

In our cohort, 148 patients underwent a total of 300 other high dose imaging procedures (272 CT and 28 NM scans), averaging 2.03 procedures per patient. DLP data for CT scans were available for only 219 exams, with a median exposure of 239 mGy·cm (IQR: 56–570). Data for NM scans were available for 25 patients, with a median of administered activity of 185 MBq (IQR: 119–217). The different types of CT and NM, with their median DLP or MBq values, are presented in Table 3. CT and NM scans accounted for only 21% of all high-dose procedures. The distribution of diagnostic cardiac catheterizations, interventional cardiac catheterizations, CTs and NM scans in the whole cohort is shown in Fig. 6.

**Table 3:** Median exposure and IQR in different types of CT and NM scans

***Computed tomography***	**N. Procedures**	**DLP, mGy**·**cm**
Chest	136	113 (41–342)
Head	29	512 (374–1027)
Heart	17	429 (75–666)
Abdomen	12	645 (481–1196)
Whole body	8	296 (93–886)
Massive facial bones	2	1361 (202–2519)
Supra-aorticangiography	1	4853
Intracranial angiography	1	3435
Shoulder/arm	1	41
Femur	1	3138
Lumbosacral spine	1	399
Whole-body PET-CT	6	473 (202–540)
Cardiac PET-CT	4	89 (58–218)
***Nuclear medicine***	**N. Procedures**	**Injected MBq**
Cardiac scintigraphy	6	217 (174–472)
Angiocardio scintigraphy	3	370 (370–370)
Pulmonary scintigraphy	2	185 (185–185)
Renal scintigraphy	6	41 (17–111)
Whole-body scintigraphy	8	185 (185–198)

*DLP* Dose‐length product, *MBq* Megabecquerel, *PET-CT* Positron emission tomography- computed tomography

### Biological samples

A total of 60 patients were enrolled in the prospective study, comprising 38 males with a median age of 7 years (IQR: 5–12). Among them, 27 patients had no previous catheterization procedures, while 33 patients had at least one previous cardiac catheterization procedure. Twenty-six patients (20 males; median age 5 years, IQR: 5–11) underwent diagnostic procedures while 34 (19 males; median age 7 years, IQR: 8–12) therapeutic ones.

The median KAP/kg value was 0.07 Gy·cm^2^/kg (IQR: 0.03–0.16).

**Fig. 7:**
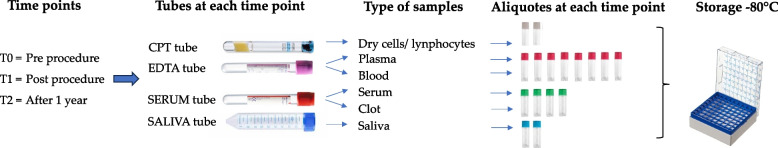
Collection of biological samples at T0 (pre-procedure), T1 (post-procedure) and T2 (after 1 year) in the prospective study

The Italian cohort collected a total of 1858 samples, including six different types of specimens: blood, plasma, serum, clot, cell pellet/lymphocytes and saliva. Figure 7 illustrates the study design for the collection of biological samples.

## Discussion

This study provides a comprehensive analysis of procedure-specific radiation data for specific interventional catheterizations in the Italian cohort of patients with CHD, contributing to the HARMONIC project. The results highlight significant variation in radiation exposure across different procedure types and age groups.

Furthermore, the study clearly shows that advancements in technology and increased operator awareness over time can effectively reduce radiation exposure in this vulnerable population. Similarly, previous studies have reported a significant reduction in radiation exposure in pediatric catheterization labs following the adoption of new X-ray technology and the implementation of optimized radiation protocols, with a focus on reducing technical changes [14–18].

As previously shown, an educational program for physicians and technicians also contributed to a significant decrease in radiation doses across various case types, particularly among infants and young children [19].

Our findings strongly emphasize that enhancing awareness and providing training to operators are crucial strategies for ensuring radiation safety not only for patients but also for staff and physicians in the catheterization laboratory, as highlighted through the Image Gently and Step Lightly campaigns [20]. Indeed, dose reduction is a collaborative effort, involving physicians, staff, medical or health physicists, and every team member plays an important role and must actively participate in managing radiation dose to optimize patient safety [10, 20].

We observed different proportions of diagnostic and therapeutic procedures across age subgroups. The higher prevalence of interventional cases observed in neonates can be attributed to the life-saving nature of these procedures, which also serve as a bridging treatment before surgery to enhance the patient's clinical status. Conversely, diagnostic tests are predominant in children aged over 15 years, serving as one of the clinical monitoring methods used in CHD patients. Additionally, apart from catheterization, we have shown that CT is the primary high-exposure examination conducted in our cohort. This observation aligns with findings from previous studies [21–23], including the recent study in the Norwegian HARMONIC cohort which showed that the CT made the most significant contribution to the radiation dose from imaging, excluding cardiac intervention [24].

Furthermore, the use of hybrid imaging through combined PET-CT examinations in our population is noteworthy.

In recent years, diagnostic PET-CT imaging has seen a substantial increase, offering both functional and anatomical details for clinical management in a single scanning session [25].

However, the use of PET-CT procedures requires special consideration regarding radiation exposure due to the combination of administered activity and X-rays from the CT, as compared to individual CT or PET examinations [26].

As a result, PET-CT studies should be employed judiciously in pediatric patients with CHD, with awareness of cumulative radiation exposure and an assessment of the overall diagnostic benefit of the scan.

Another crucial outcome of our study is the collection of large number of biological samples contributing to the Harmonic biobank, with the goal of enhancing our understanding on radiation-related biological changes following pediatric exposure to ionizing radiation [13].

Previous biological studies from our group demonstrated that cardiac catheterization procedures lead to increased short-term and long-term chromosomal DNA damage [21, 26, 27], as well as the shortening of leukocyte telomere length, reduction of mitochondrial DNA copy number (mtDNAcn) and dysregulation of oncogenic microRNAs [28, 29].

Nowadays, Harmonic biobank will enhance the epidemiological approach of the project, offering a unique opportunity to achieve a more comprehensive understanding of the underlying biological and cellular mechanisms related to pediatric radiation exposure [13].

Doses to individual organs will be estimated using dose indicators recorded at the time of examination through Monte Carlo simulations and an anatomically realistic computational phantom model [12]. This approach will allow the analysis of the dose–response relationship with biomarkers and the evaluation of an individual's physiological response. Additionally, the longitudinal design will permit the assessment of the early biological response to IR exposure, the persistence of biological changes over time, and their predictive ability for long-term damage before the onset of overt clinical disease or the development of cancer. Since contrast media may increase DNA damage by 50%, we will also assess the combined effect of the concentration of iodinated contrast and IR exposure [30, 31].

However, we acknowledge several limitations in our Italian cohort that warrant consideration. First, conventional radiography examinations were not included considering that the dose contribution per examination was low and sometimes the exam was not recorded. Second, we cannot completely exclude the possibility that exposure to medical radiation may have occurred at outside healthcare facilities. Third, this is a cohort study conducted at a single large children's Italian hospital, with a strong emphasis on employing radiation dose-reduction protocols, and thus these radiation exposure data may not necessarily be generalizable to other pediatric care hospitals in Italy.

## Conclusions

In conclusion, the Italian Harmonic cohort provides comprehensive information on typical levels of doses for pediatric catheterization procedures. The institution’s radiological protection strategy has contributed to a reduction in radiation dose over time, emphasizing the importance of the radiological awareness within the clinical community as an effective strategy for enhancing the safety of patients and staff [32]. Biological samples provide a valuable resource for future research, offering the opportunity to identify potential early biomarkers for health surveillance and personalized risk assessment.

## Data Availability

The datasets generated and/or analyzed during the current study are not publicly available because the data was primarily collected for project HARMONIC and cannot be distributed to other parties.

## Abbreviations

Cath: Cardiac catheterization
CHD: Congenital heart disease
CT: Computed tomography
DLP: Dose‐length product
FT: Fluoroscopic time
IQR: Interquartile range
IR: Ionizing radiation
KAP: Kerma area product
MBq: Megabecquerel
NM: Nuclear medicine
PET-CT: Positron emission tomography- computed tomography

## References

[1] van der Linde D, Konings EE, Slager MA, Witsenburg M, Helbing WA, Takkenberg JJ (2011). Birth prevalence of congenital heart disease worldwide: a systematic review and meta-analysis. J Am Coll Cardiol.

[2] Campolo J, Annoni G, Giaccardi M, Andreassi MG (2022). Congenital heart disease and the risk of cancer: an update on the genetic etiology, radiation exposure damage, and future research strategies. J Cardiovasc Dev Dis.

[3] Modan B, Keinan L, Blumstein T, Sadetzki S (2000). Cancer following cardiac catheterization in childhood. Int J Epidemiol.

[4] McLaughlin JR, Kreiger N, Sloan MP, Benson LN, Hilditch S, Clarke EA (1993). An historical cohort study of cardiac catheterization during childhood and the risk of cancer. Int J Epidemiol.

[5] Cohen S, Liu A, Gurvitz M, Guo L, Therrien J, Laprise C (2018). Exposure to low-dose ionizing radiation from cardiac procedures and malignancy risk in adults with congenital heart disease. Circulation.

[6] Harbron RW, Chapple CL, O'Sullivan JJ, Lee C, McHugh K, Higueras M (2018). Cancer incidence among children and young adults who have undergone x-ray guided cardiac catheterization procedures. Eur J Epidemiol.

[7] Stern H, Seidenbusch M, Hapfelmeier A, Meierhofer C, Naumann S, Schmid I (2020). Increased cancer incidence following up to 15 years after cardiac catheterization in infants under one year between 1980 and 1998-a single center observational study. J Clin Med.

[8] Abalo KD, Malekzadeh-Milani S, Hascoët S, Dreuil S, Feuillet T, Cohen S (2021). Exposure to low-dose ionising radiation from cardiac catheterisation and risk of cancer: the COCCINELLE study cohort profile. BMJ Open.

[9] Abalo KD, Malekzadeh-Milani S, Hascoët S, Dreuil S, Feuillet T, Damon C (2023). Lympho-hematopoietic malignancies risk after exposure to low dose ionizing radiation during cardiac catheterization in childhood. Eur J Epidemiol.

[10] Andreassi MG, Picano E (2014). Reduction of radiation to children: our responsibility to change. Circulation.

[11] Cohen S, Gurvitz MZ, Beauséjour-Ladouceur V, Lawler PR, Therrien J, Marelli AJ (2019). Cancer risk in congenital heart disease-what is the evidence?. Can J Cardiol.

[12] Harbron RW, Thierry-Chef I, Pearce MS, Bernier MO, Dreuil S, Rage E, et al. The HARMONIC project: study design for the assessment of radiation doses and associated cancer risks following cardiac fluoroscopy in childhood. J Radiol Prot. 2020;40(4):1074–90. 10.1088/1361-6498/aba66d 32668420

[13] Andreassi MG, Haddy N, Harms-Ringdahl M, Campolo J, Borghini A, Chevalier F (2023). A longitudinal study of individual radiation responses in pediatric patients treated with proton and photon radiotherapy, and interventional cardiology: Rationale and research protocol of the HARMONIC Project. Int J Mol Sci.

[14] Borik S, Devadas S, Mroczek D, Lee KJ, Chaturvedi R, Benson LN (2015). Achievable radiation reduction during pediatric cardiac catheterization: How low can we go?. Catheter Cardiovasc Interv.

[15] Mauriello DA, Fetterly KA, Lennon RJ, Reeder GS, Taggart NW, Hagler DJ (2014). Radiation reduction in pediatric and adult congenital patients during cardiac catheterization. Catheter Cardiovasc Interv.

[16] Lamers LJ, Moran M, Torgeson JN, Hokanson JS (2016). Radiation reduction capabilities of a next-generation pediatric imaging platform. Pediatr Cardiol.

[17] Amdani SM, Ross RD, Webster PA, Turner DR, Forbes TJ, Kobayashi D (2018). Reducing radiation exposure by lowering frame rate in children undergoing cardiac catheterization: A quality improvement study. Congenit Heart Dis.

[18] Patel C, Grossman M, Shabanova V, Asnes J (2019). Reducing radiation exposure in cardiac catheterizations for congenital heart disease. Pediatr Cardiol.

[19] Verghese GR, McElhinney DB, Strauss KJ, Bergersen L (2012). Characterization of radiation exposure and effect of a radiation monitoring policy in a large volume pediatric cardiac catheterization lab. Catheter Cardiovasc Interv.

[20] Hill KD, Frush DP, Han BK, Abbott BG, Armstrong AK, DeKemp RA (2017). Radiation safety in children with congenital and acquired heart disease: a scientific position statement on multimodality dose optimization from the image gently alliance. JACC Cardiovasc Imaging.

[21] Ait-Ali L, Andreassi MG, Foffa I, Spadoni I, Vano E, Picano E (2010). Cumulative patient effective dose and acute radiation-induced chromosomal DNA damage in children with congenital heart disease. Heart.

[22] Johnson JN, Hornik CP, Li JS, Benjamin DK, Yoshizumi TT, Reiman RE (2014). Cumulative radiation exposure and cancer risk estimation in children with heart disease. Circulation.

[23] Glatz AC, Purrington KS, Klinger A, King AR, Hellinger J, Zhu X (2014). Cumulative exposure to medical radiation for children requiring surgery for congenital heart disease. J Pediatr.

[24] Afroz S, Østerås BH, Thevathas US, Dohlen G, Stokke C, Robsahm TE (2023). Use of ionizing radiation in a Norwegian cohort of children with congenital heart disease: imaging frequency and radiation dose for the Health Effects of Cardiac Fluoroscopy and Modern Radiotherapy in Pediatrics (HARMONIC) study. Pediatr Radiol.

[25] Meyer Z, Fischer M, Koerfer J, Laser KT, Kececioglu D, Burchert W (2016). The role of FDG-PET-CT in pediatric cardiac patients and patients with congenital heart defects. Int J Cardiol.

[26] Hosono M, Takenaka M, Monzen H, Tamura M, Kudo M, Nishimura Y (2021). Cumulative radiation doses from recurrent PET-CT examinations. Br J Radiol.

[27] Andreassi MG, Ait-Ali L, Botto N, Manfredi S, Mottola G, Picano E (2006). Cardiac catheterization and long-term chromosomal damage in children with congenital heart disease. Eur Heart J.

[28] Vecoli C, Borghini A, Foffa I, Ait-Ali L, Picano E, Andreassi MG (2016). Leukocyte telomere shortening in grown-up patients with congenital heart disease. Int J Cardiol.

[29] Borghini A, Campolo J, Annoni G, Giuli V, Sicari R, Peretti A (2023). Cancer risk in patients with congenital heart disease exposed to radiation from cardiac procedures. J Am Coll Cardiol.

[30] Berrington de Gonzalez A, Kleinerman RA. CT scanning: is the contrast material enhancing the radiation dose and cancer risk as well as the image? Radiology. 2015;275(3):627–9. 10.1148/radiol.2015150605 PMC4553968 25997129

[31] Van Cauteren T, Tanaka K, Belsack D, Van Gompel G, Kersemans V, Jochmans K (2021). Potential increase in radiation-induced DNA double-strand breaks with higher doses of iodine contrast during coronary CT angiography. Med Phys.

[32] Picano E, Vañó E, Rehani MM, Cuocolo A, Mont L, Bodi V (2014). The appropriate and justified use of medical radiation in cardiovascular imaging: a position document of the ESC Associations of Cardiovascular Imaging, Percutaneous Cardiovascular Interventions and Electrophysiology. Eur Heart J.

